# GDF11 Implications in Cancer Biology and Metabolism. Facts and Controversies

**DOI:** 10.3389/fonc.2019.01039

**Published:** 2019-10-15

**Authors:** Arturo Simoni-Nieves, Monserrat Gerardo-Ramírez, Gibrán Pedraza-Vázquez, Lisette Chávez-Rodríguez, Leticia Bucio, Verónica Souza, Roxana U. Miranda-Labra, Luis E. Gomez-Quiroz, María Concepción Gutiérrez-Ruiz

**Affiliations:** ^1^Posgrado en Biología Experimental, DCBS, Universidad Autónoma Metropolitana-Iztapalapa, Mexico City, Mexico; ^2^Laboratorio de Fisiología Celular y Biología Molecular, Departamento de Ciencias de la Salud, Universidad Autónoma Metropolitana-Iztapalapa, Mexico City, Mexico; ^3^Laboratorio de Medicina Experimental, Unidad de Medicina Translacional, Instituto de Investigaciones Biomédicas, UNAM/Instituto Nacional de Cardiología Ignacio Chavez, Mexico City, Mexico

**Keywords:** GDF11, PCSK5, cancer, liver, HCC, metabolism

## Abstract

Growth Differentiation Factor 11 (GDF11), a member of the super family of the Transforming Growth Factor β, has gained more attention in the last few years due to numerous reports regarding its functions in other systems, which are different to those related to differentiation and embryonic development, such as age-related muscle dysfunction, skin biology, metabolism, and cancer. GDF11 is expressed in many tissues, including skeletal muscle, pancreas, kidney, nervous system, and retina, among others. GDF11 circulating levels and protein content in tissues are quite variable and are affected by pathological conditions or age. Although, GDF11 biology had a lot of controversies, must of them are only misunderstandings regarding the variability of its responses, which are independent of the tissue, grade of cellular differentiation or pathologies. A blunt fact regarding GDF11 biology is that its target cells have stemness feature, a property that could be found in certain adult cells in health and in disease, such as cancer cells. This review is focused to present and analyze the recent findings in the emerging research field of GDF11 function in cancer and metabolism, and discusses the controversies surrounding the biology of this atypical growth factor.

## Introduction

On May 2013 the research groups, led by doctors Amy J. Wagers and Richard T. Lee, published outstanding work suggesting that the growth differentiation factor 11 (GDF11) could be a good candidate for the age-related heart hypertrophy reversion observed in the model of heterochronic parabiosis (1). One year later, on May 2014, Science journal published a couple of works by the same research team at Harvard University, unveiling that systemic injection of the GDF11 reverses age-related dysfunction in skeletal muscle (2) and vascular and neurogenic function in the brain (3). Both reports were astonishing, particularly because myostatin, also known as GDF8, shares high structural homology with GDF11, but GDF8 induces exactly the contrary effect, muscle growth inhibition (4). At that moment, GDF11 was called “the rejuvenation factor,” a term taken by a commentary note published by Jocelyn Kaiser in the same number of the Science journal (5), and Karoline E. Brun published another similar commentary in Cell journal entitle “GDF11 and the Mythical Fountain of Youth” (6).

The findings, beyond this unfortunate motto, revealed that GDF11 could exert functions in adult systems, in addition of those characterized in embryonic and fetal tissues. The works by the groups of doctors Wagers and Lee provided evidence that the main target cells are those with certain stemness phenotype, such as the satellite cells in the muscle, which are the progenitor ones for new functional muscle cells.

If GDF11 targets cells with stemness capacity, then many cancer cells should be targeted by this growth factor.

Many cancer cells gain stemness capacity and this correlates with aggressiveness and poor prognosis. The findings raised by the group of doctors, Wagers and Lee, position cancer cells as a target of GDF11 since they proved that stemness is a key condition for GDF11 effect. However, the results could be opposite depending of the cancer cell origin, metabolic status, or the stage of the cancer. We must wait for incoming works in the next few years, perhaps months, revealing a more precise mechanism regarding these apparent controversies in cancer and metabolism.

This work is focused to review the general knowledge of GDF11, and its functions in cancer biology and metabolism, taking into consideration recent findings in the specialized literature and in the public databases and scientific on-line resources.

## GDF11 an Atypical TGF-β Family Member

GDF11 (also known as Bone Morphogenetic Protein, BMP11), is a member of the super family of the Transforming Growth Factor beta (TGF-β) and a subfamily of the BMP which is widely secreted in many species, including mouse, rat and human, and it is accepted as a key factor in embryo development, particularly in the anterior/posterior patterning (7–9).

GDF11 was identified by McPherron et al. in 1999, who cloned the human and mouse GDF11 and characterized its function in pattering the axial skeleton (9). Two years prior, the same group also discovered and characterized the GDF8 (10).

**Figure 1:**
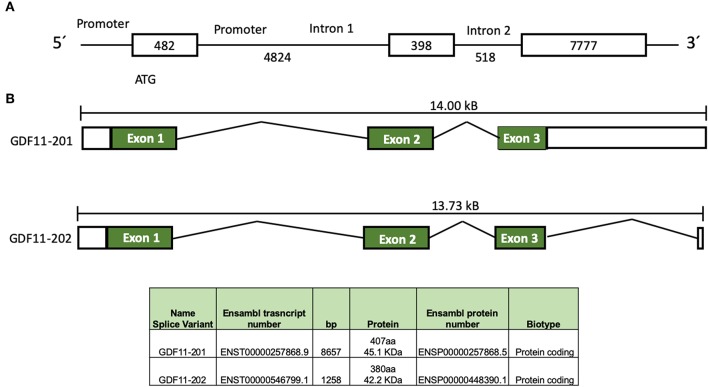
Schematic representation of GDF11 gene and mRNA. **(A)** Gdf11 gene and **(B)** Gdf11 transcripts and table with the two transcripts specifications according to Ensembl (www.ensembl.org, ENSG00000135414.9) and Jeanplog 2014.

In humans, GDF11 gene is located in chromosome 12 (12q13.2, forward strand, Ensembl accession number: ENSG00000135414). Two splice variants products have been identified, according to Ensembl (Figure 1), the first one, GDF11-201 is a 8657 bp RNA, formed by three coding exons, generating a 407 amino acids protein, and the second one, GDF11-202, is a 1,258 bp, formed by three exons generating a 380 amino acids protein (11). Jeanplong (12) reported another RNA splice variant determined as GDF-11ΔEx11, characterized by the absence of exon 1, and composed for exon 2 and 3 with transcriptional initiation in intron 1 (4,701 bp). It is predicted this variant could be regulated by transcription factors, such as some myogenic factors (MRF, Myf5, MyoD, Myogenin, and MRF4), Pax3, NF1, AP1, among others (12), suggesting that it could be involved in muscle development and/or repair as reported in other work (2). Interestingly, the promoter of GDF11 could also be activated by trichostatin A (13), an inhibitor of histone deacetylases (HDAC), suggesting a clear epigenetic regulation of the GDF11 gene expression; HDAC3 regulates zebrafish liver development by modulating GDF11. The overexpression of HDAC3 increases liver size, while the increase of GDF11 expression induces a small size liver; interestingly, the knockdown of GDF11 did not induce any relevant change in liver morphology. The role of HDAC3 in GDF11 function in liver development is likely a direct control over the hepatocyte precursor (hepatoblast) proliferation, as observed in HCC-derived cells (14), but this must be deeply addressed.

**Figure 2:**
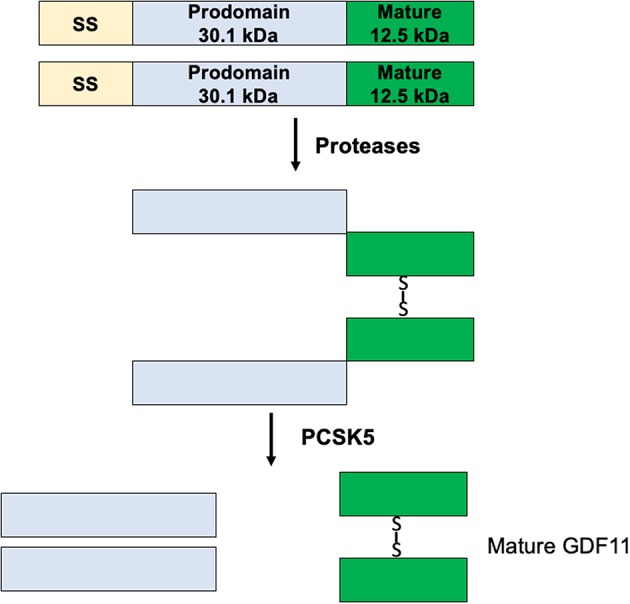
Maturation process of GDF11.

GDF11 mRNA is translated in a precursor protein (Figure 2), which is processed by specific proteases generating the mature GDF11 (C-terminal, 12.5 kDa) and the pro-domain (N-terminal, 30.1 kDa). GDF11 shares 89% amino acid sequence homology with GDF8, however GDF8 expression in human tissues is restricted to cardiac and skeletal muscle (1), while GDF11 is practically expressed in all tissues (15). Although there is high homology between mature GDF8 and GDF11, the pro-domains of both proteins share only 54% homology. The pro-domain is fundamental for proper protein folding, disulfide bond formation and exportation of the homodimers (16), suggesting differences in post-translational process.

The protein convertase subtilisin/kexin 5 (PCSK5) is one of the main acting proteins on GDF11, activating the mature GDF11 by proteolytic process at basic sites of the pro-domain (17). The elimination of PCSK5 in the mouse embryo was associated with abnormal expression of Hlxb9 and Hox genes, two well-known GDF11 target genes, generating defects in the anteroposterior patterning and strongly proposing a relationship with GDF11 functions (7, 8).

In humans, GDF11 is expressed in practically all tissues, but is particularly relevant in the brain (hippocampus), the kidneys, the endometrium, and the heart muscle; while the liver is the organ with the lowest expression (1, 15, 18).

## The Signal Transduction

**Figure 3:**
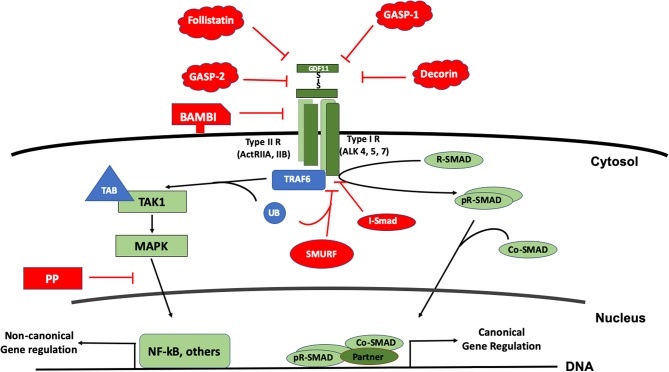
The signal transduction of GDF11. The figure displays the canonical signal transduction mediated by R-SMAD (SMAD 2/3, SMAD 1,5,8), assisted by the Co-SMAD (SMAD4). The signal could inhibited by inhibitory SMAD (I-SMAD) 6 or 7 or by the SMAD specific E3 ubiquitin protein ligase (SMURF). Extracellularly, GDF11 could be inhibited by the BMP and activin membrane-bound inhibitor (BAMBI), or the action of proteins such as Follistatin, Decorin, and GDF-associated serum protein-1 (GASP-1) and GASP-2. The non-canonical pathway is driven particularly by the Mitogen Activated Proteins Kinases (MAPK), signaling continues to the tumor necrosis factor receptor-associated factor (TRAF) 2 or 6; and TGF-β activated kinase 1 (TAK1), which in addition uses the TAK1 binding protein (TAB) 1 and one of both 2 or 3. Non-canonical regulation could influence the nuclear factor kappa B (NF-kB) among others, and the inhibition of this pathway could be blocked by protein phosphatases (PP).

As a member of the BMP family, GDF11 uses the canonical receptors and the SMAD proteins for signaling. The GDF11 dimer (a disulfide-linked homodimer of carboxy-terminal fragments) binds the activin receptors type II A or B (ActRIIA, ActRIIB), proteins with serine/threonine kinase activity; leading to the recruitment and transphosphorylation of two type I serine/threonine kinase receptors, also known as activin-like kinase receptors (ALK), particularly the 4, 5, or 7 (19, 20). The activated ALK receptor phosphorylates and activates the receptor-regulated SMAD (R-SAMD). GDF11 particularly transduces by using SMAD2 and 3 (14, 21), and some reports also propose the participation of SMAD1, 5 and 8 (22). The R-SMAD dimer recruits the co-SMAD, SMAD4, to form a trimeric complex, which eventually translocates to the nucleus for gene expression regulation (23). Although the signal transduction of the TGF-β family might seem simple, it is highly regulated by extracellular and intracellular mechanisms. Inside the cell, the regulation can occur at the membrane or in the cytosol, during nuclear translocation and DNA biding, at this level, is a tetrameric complex because the interaction with a fourth protein component or partner (24) (Figure 3).

GDF11 can also transduce by non-canonical pathways. Mitogen activated protein kinase (MAPK) is perhaps the main non-SMAD pathway controlled by the growth factor, activating routes such as p38, AKT, and JNK (25, 26), however, in some cases, inhibiting the activation of JNK or NF-κB (27) depending of the cell lineage. Further, it has been described that the family can also transduce by MAPKKK7 [also known as TGF-β activated kinase 1 (TAK1)] via MEK6 (28–30). TAK1 is part of a signaling complex formed by TAK1 binding protein 1 (TAB1) and with either TAB2 or TAB3 (31). TAK1 complex follows an intricate mechanism of activation involving the tumor necrosis factor receptor-associated factor (TRAF) 2 or 6, adaptor proteins with non-conventional activity of E3 ubiquitin ligase. TRAF proteins exert regulation over TAB2 or 3. Finally, the autophosphorylation of TAK1 leads to the activation of its downstream targets, particularly members of the MAPK and NF-κB signaling pathways (32).

Negative regulation of the GDF11-mediated signaling can also occur at different levels. Extracellularly, GDF11 can be negatively regulated by the interaction with many proteins such as follistatin (33, 34), GDF-associated serum protein-1 (GASP-1), GASP-2 (35), decorin and follistatin-like 3, among others (4). Follistatin, a secreted glycoprotein, binds GDF11 and inhibits its interaction with ActRIIB. Follistatin is the main extracellular inhibitor of GDF11, and is transcriptionally regulated by the same GDF11 signaling, indicating that the signal transduction is restricted by a negative feedback mechanism (36).

The BMP and activin membrane-bound inhibitor (BAMBI), a co-receptor that is not functional due to it lacks cytosolic domain, has been suggested to be another negative regulator in plasma membrane, but that still remains to be confirmed (19).

In the cytosol, GDF11 follows the canonical negative regulation of the family. It has been reported that GDF11 is regulated by SMAD7 (37) and SMAD6 (19). The SMAD specific E3 ubiquitin protein ligase 2 (SMURF2) also displays negative regulation of the signaling pathway (28). Negative-regulation of the non-canonical pathway is driven by specific protein phosphatases (PP), such as PPC1, among others.

## Development and Aged-Related Function

Although GDF11 was identified in 1999 (9), as previously mentioned, in 2014 the growth factor was transiently located in the “*Sancta sanctorum*” of the “miraculous” molecule, when the laboratory of Amy Wagers (2) reported that GDF11 was responsible for the skeletal muscle regeneration in mice heterochronic parabiosis. A profound controversy arose regarding the rejuvenating property of GDF11; some groups stated that this property is displayed by the growth factor (1–3, 38), while others reported the opposite effect (39–42), as previously mentioned. To have a good point of view regarding this debate, we suggest a deep view of cited works and commentaries regarding the controversy (3, 5, 6, 19, 42, 43).

Regardless of this disagreement, there is no doubt about the GDF11 function in differentiation and embryonic development, particularly in anterior/posterior axial skeleton (9) and brain function (44), which are nicely reviewed elsewhere (19, 45, 46).

## GDF11 vs. GDF8 and the Race For the Discovery of the Rejuvenation Properties

GDF11 and GDF8 are close related members of the activins subclass in the TGF-β superfamily. Sharing 90% of their amino acid sequence (38, 47), these two proteins have been a technical challenge for antibody manufacturers and, therefore, protagonists of one of the most controversial studies in recent years (29, 39, 48–50), regarding to the issue of GDF11 being the protein responsible for “rejuvenation” of aged organisms (1, 2), as previously mentioned.

The race from the discovery of the rejuvenation properties of GDF11 to the following debate of the antibody specificity led to a deeper structural analysis of these proteins and the interaction with their receptor. Due to the similarities of ~90% of sequence identity of the C-terminal signaling domain between GDF11 and GDF8, their mature form is nearly identical, which causes these proteins to share the same activin type II receptor (38).

Although they are indeed similar in their monomeric form, in fact these proteins are thought to have opposite functions, where GDF11 works as a muscle generator in embryogenesis (9) but GDF8 acts as a muscle mass inhibitor (10, 51), which may be the result of the final homodimer structure. Thus, it is important to understand that the GDF11 and GDF8 homodimer formation leads to a different conformation that allows them to interact with the same receptor in a unique and specific way. It is reported that both homodimers are linked by a single disulfide bond in an antiparallel conformation, but the flexibility in the relative orientations generated by the differences in their structure are determinant for the quaternary structure variations that lead to a distinctive biological response (47, 52).

It has also been reported that GDF11 has a stronger affinity for the receptor than GDF8 (38) and that it is more dependent on direct receptor contacts (53), but there is also an issue with crystal structures of both proteins. Human myostatin alone has not been reported and the available structures are bound to extracellular antagonists (follistatin and follistatin-like 3) (54, 55), which have been compared to a small-angle X-ray scattering (SAXS) analysis to determine the mechanism of activation (52). On the other hand, human GDF11 structure has been resolved in recent years (47), thus, it is possible to discover the real impact of the structure of both proteins in future, at which point we can begin to uncover exactly what makes the responses so different.

## GDF11 Effects in Cancer Biology

An emerging field of research is the impact of GDF11 in cancer biology. Most of the cancer cells, particularly those with high aggressiveness, retain or recover stemness capacity, placing them as a potential target of GDF11 (14, 23).

There exist some controversies in cancer biology as well; in some cases GDF11 induces clear tumor suppressive properties (14, 23), and in others it is the opposite (56, 57). Once again, the versatility displayed by this growth factor depends of cell progeny, grade of differentiation or transformation.

## Liver Cancer

We recently published work describing how GDF11 induces tumor suppressive properties in human hepatocellular carcinoma-derived cells, Huh7 and Hep3B cell lines, restricting spheroid formation and clonogenic capacity, an effect that is also observed in other liver cancer cell lines (SNU-182, Hepa1-6, and HepG2), decreasing proliferation, motogenesis, and invasion. These characteristics were associated with transcriptional repression of cyclin D1 and A, and the overexpression of p27 (14). GDF11 effects, on hepatic cell proliferation, have been found in liver development, where GDF11 targets the hepatoblast, the hepatocyte precursor (13, 58).

Remarkably, the invasion experiments using the chick embryo chorioallantoic membrane (CAM) model (14, 59) revealed a static phenotype in Huh7 cells treated for 72 h with GDF11 (50 ng/ml), an outcome well-correlated with a decrease in cell migration and proliferation. Furthermore, GDF11 treated cells were incapable of sustaining colony and sphere capacity in the absence of GDF11, up to 5 days, indicating that the effect of GDF11 on self-renewal capacity is not transient, suggesting a reprogramming effect.

Similar results were obtained in the hepatoblastoma cell lines, HepG2 and SMMC-7721: the treatment with GDF11 up to 72 h reduced cell viability. Although SMMC-7721 cells are probably a HELA-derivative cell line, the effect was also present (60). This report also provides preliminary evidence that the expression of GDF11 was significantly lower in cancerous tissue rather than in normal liver.

Outstandingly, GDF11 was capable of decreasing aggressiveness-associated markers in Huh7 and Hep3B cells, producing a deregulation in the expression of *Epcam, promo1* (CD133), *cd24, and ck19*, that was associated with the repression of Snail and N-cadherin, and the overexpression of occluding and E-cadherin, strongly indicating a mesenchymal to epithelial transition (14).

It is interesting that, under normal conditions, liver cells, which are the poorest in GDF11 production, are highly responsive to GDF11 in the context of cancer could be relevant in terms of a possible use of GDF11 for treatment. The work by Gerardo-Ramírez clearly showed that all HCC cells used in the study responded to the exogenous GDF11 treatment, decreasing all aggressiveness-associated markers. Interestingly, the effects in HCC cells were differentiated, and it was dependent of the stemness capacity, being more responsive to Hep3B cells, which express fewer stemness markers compared to Huh7 cells. Supporting this statement, in liver development GDF11 has been related to inhibition of liver growth, mainly targeting proliferation of hepatoblast, the cell precursor or mature hepatocytes by a mechanism involving HDAC3, which inhibits the expression of GDF11 as proven by Farooq and collaborators (58). This work clearly demonstrates that GDF11 targets hepatic cells with stemness features, not necessarily those observed in cancer, but in the normal liver, particularly in development.

## Breast Cancer

Similarly, Bajikar et al. (23) identified a tumor-suppressive role of GDF11 in a triple-negative breast cancer (TNBC). These cells, under 3D culture, heterogeneously express GDF11 and very low levels of GDF8, as well as the main canonical receptors, such as ALK4, and ALK5, among other protein machinery required for a proper signal transduction. This clearly indicates that breast epithelial cells express the required components to recognize GDF11 as an autocrine or paracrine stimulus (23). GDF11 also induced a decrease in number and size of the spheroids and generated more-compacted structures by the increment in E-cadherin, as observed in liver cancer cell lines, and GDF11 treatment induces a cell-cell adhesion preventing metastasis phenomena (14, 23).

Authors also found a defective GDF11 maturation and secretion in seven of nine studied TNBC cell lines. The linker was the convertase PCSK5, in which a deficiency was found in the TNBC cells, inducing the extracellular accumulation of the immature proGDF11 and, for instance, loss in the bioactivity of GDF11. This mechanism was also observed in mice; the lack of Pcsk5 in Apc^min/+^ animals (61) increases adenocarcinoma formation in the small intestine, decreasing the survival (23, 62), which demonstrates a clear function in tandem of GDF11 and PCSK5 to induce the tumor suppressive properties. In fact, the restoration of the PCSK5 activity in the TNBC cells suppresses lung metastasis (23).

Another work by Wallner et al. (63) revealed that super-physiological levels of GDF11 (2 μg/ml) could provide advantages in chemotherapy in breast adenocarcinoma, inducing a decrement in the migrative capacity of MCF-7 cells in a scratch assay. Similar findings were observed in the presence of follistatin (2 μg/ml), while GDF8 (2 μg/ml) induced cell death at the same time. This study also showed that GDF11 is expressed in low grade adenocarcinoma tissue (G1), but lower levels in G3 tissue were found, and it was correlated with high expression of follistatin in G1, suggesting an inhibitory effect of GDF11 at higher levels of differentiation, which is consistent with the idea that high aggressiveness in cancer associates with less GDF11 function, confirming the tumor suppressive capacity of GDF11.

## Pancreatic Cancer

Pancreatic cancer (PC) represents one of the most lethal cancers worldwide (64). It has been reported that GDF11 is down-regulated in PC tissue, compared with surrounding tissue, and pancreatic cell lines exhibit a low expression of the growth factor (65). This group also reported that, in a cohort of 63 PC patients, those with high GDF11 expression had significantly better survival rates in comparison with those with low GDF11 expression. These effects were related to decreased proliferation, migration and invasion, and these observations are in agreement with those reported in HCC and TNBC. GDF11 is also capable of inducing apoptosis in PC cell lines (65).

**Figure 4:**
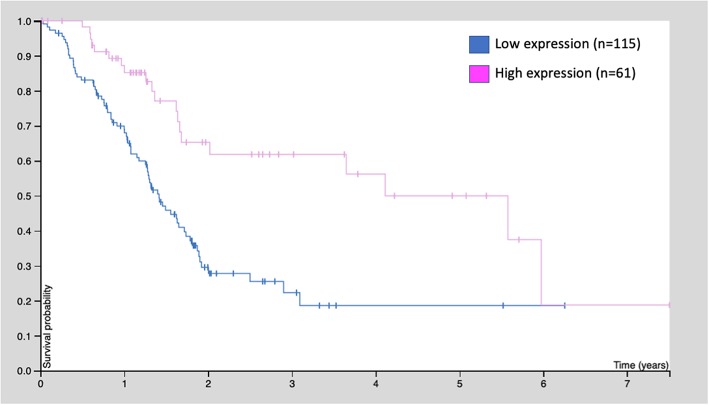
Kaplan-Meier survival curve of 176 patients with pancreatic cancer. Patients were classified as low GDF11 expression and high GDF11 expression, *p* < 0.001, according to the human protein atlas (www.proteinatlas.org/ENSG00000135414-GDF11/pathology).

Similarly, the human protein atlas (https://www.proteinatlas.org) provides evidence from 176 patients: those with high GDF11 expression (*n* = 61) exhibited better survival rates, compared with those with low expression (*n* = 115, *p* < 0.001) (Figure 4). These observations strongly suggest that GDF11 could also exert tumor suppressive properties that should be deeply addressed to gain confidence, particularly the effect of exogenous active GDF11 (18).

Interestingly, another member of the family GDF15 is directly correlated with poor survival in PC patients, and it is proposed as a better marker than CA-125 (66), again raising the atypical functions of this growth factor.

As observed in HCC, in PC, the targets of GDF11 are poorly differentiated cells. In the mouse embryo, GDF11 is expressed in the pancreatic epithelium, at embryonic day E12-E14 (67), as it happens in the liver, but in GDF11^−/−^ animals the pancreas size are 2-fold smaller than wild type.

In the context of the educated guess that cells with some stemness phenotype respond to GDF11, even in cancer, it has been proven that GDF11 negatively regulates NGN3^+^ progenitor cells and GDF11 induces β-cell differentiation (68), supporting the role of GDF11 in metabolism. Under this context, GDF11 exerts its functions in pancreatic cells with stemness phenotype.

## Colorectal Cancer

In 130 patients with colorectal cancer (CRC), the expression of GDF11 was significantly higher compared with normal tissue (56). The classification of the patient cohort in low and high GDF11 expression revealed that those patients with high levels of GDF11 showed a higher frequency of lymph node metastasis, more deaths and lower survival. The study suggests that GDF11 could be a prognostic biomarker in patients with this disease.

It is known that lymphangiogenesis is a fundamental phenomenon for colorectal cancer dissemination (69). Recently, Ungaro and collaborators reported that the microenvironment in the lymphatic vessels provides support to the tumor-derived cells by manipulating the production of extracellular matrix proteins and soluble factors, such as cytokines and growth factors (70). Whole transcriptomic analysis addressed by RNA-seq of isolated human intestinal lymphatic endothelial cells (HILEC) from surgically resected CRC and healthy corresponding controls, revealed that among those genes differentially expressed, GDF11 was observed as a significant increment with high statistical confidence. CACO-2 cells demonstrated high proliferation in co-culture with CRC-HILEC, but the GDF11 silencing by siRNA abrogated this effect indicating a tumor promotion role of GDF11 in CRC. Interestingly, GDF11 was expressed not only in lymphatic vessels in CRC, but also in normal tissue (69). The study also provides evidence of a direct correlation of GDF11 expression and tumor stage, confirming in this particular cancer that GDF11 expression could be a marker of tumor progression (70), and also raises mechanistic evidence that microenvironment in the lymphatic vessel could play a pivotal role in metastasis by local production of GDF11.

## Other Types of Cancer

Some reports have pointed to the pro-tumorigenic properties of GDF11, with major or minor confidence of rigorous scientific approach.

In oral squamous cell carcinoma, Qin and coauthors (57) showed that in a small patient cohort GDF11 expression is positively correlated with aggressiveness, finding a higher expression in metastatic oral cancer (*n* = 19) in comparison with non-metastatic oral cancer (*n* = 15). Authors also sustain that GDF11 induced epithelial to mesenchymal transition by downregulating epithelial markers such as E-cadherin, and the overexpression of vimentin or metalloproteinase 9.

In uveal melanoma, GDF11 expression was significantly upregulated compared with surrounding tissue, the expression was higher in stage IV and substantially greater in the deceased cases regarding living cases (71). The multivariate analysis confirmed that GDF11 is an independent prognostic indicator of unfavorable overall survival.

## GDF11 and PCSK Mutations

**Figure 5:**
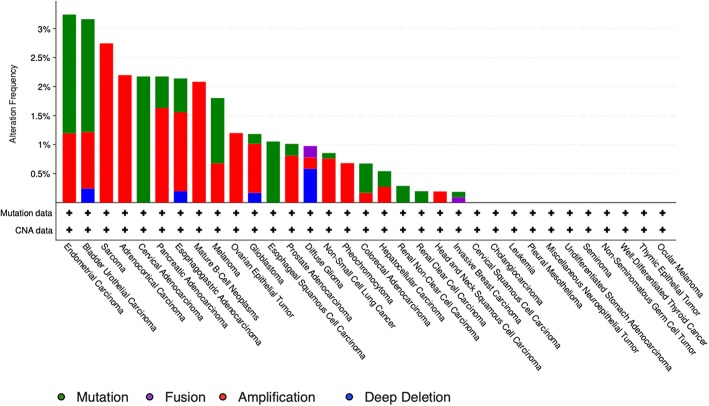
Genomic alterations in Gdf11 gene. Alteration frequency by type of cancer according to cBioportal for cancer genomics (https://www.cbioportal.org).

The study by Liu et al. (71) also showed that no relevant mutations were observed in the GDF11 gene in fact. The cBioportal for cancer genomics web site (https://www.cbioportal.org) indicates that GDF11 is altered in 1% of database patients. Figure 4 shows the alteration frequency in *Gdf11* gene in some cancers, and Figure 5 depicts the number of somatic mutations, most of which are missense (72). It seems that mutations in the Gdf11 gene are not the main consequence in those cancers where GDF11 is a prognostic factor, which increases research interest in transcriptional and post-translational regulation.

**Figure 6:**
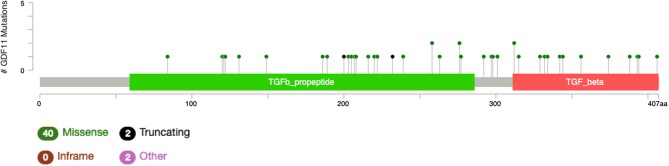
Number of mutations in Gdf11 gene. According to cBioportal for cancer genomics (https://www.cbioportal.org). RefSeq: NM_005811. Ensembl ENST00000257868. CCDS: CCDS8891. UniProt:GDF11_HUMAN. Somatic Mutations Frequency: 0.4%. Forty missense mutations. Two truncating, 0 inframe, 2 other.

**Figure 7:**
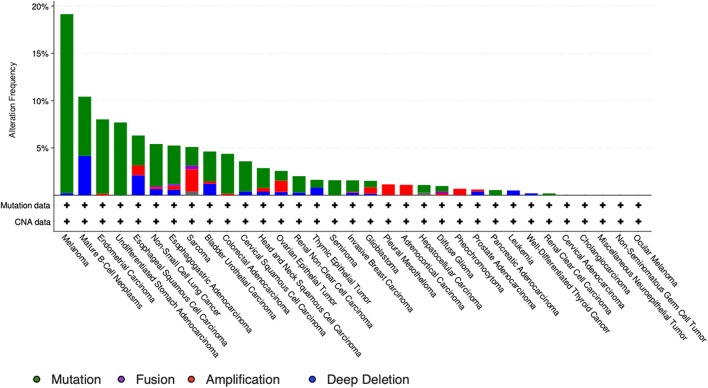
Genomic alterations in Pcsk5 gene. Alteration frequency by type of cancer according to cBioportal for cancer genomics (https://www.cbioportal.org).

**Figure 8:**
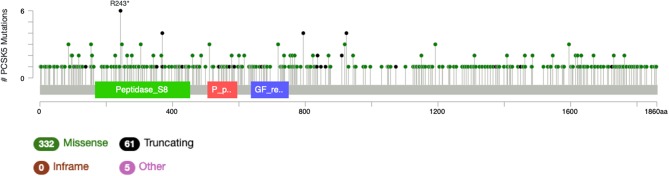
Number of mutations in Pcsk5 gene. According to cBioportal for cancer genomics (https://www.cbioportal.org). RefSeq: NM_001190482. Ensembl ENST00000545128. CCDS: CCDS55320. UniProt: PCSK5_HUMAN. Somatic Mutations Frequency: 2.8%. Three hundred thirty-two missense mutations, 61 truncating, 0 inframe, 5 others.

It is particularly relevant to consider the convertase PCSK5, a key regulator of GDF11 activity. Pcsk5 gene presents a high frequency of genomic alterations in 3% of the patients, according to cBioportal, being particularly relevant in melanoma, endometrial carcinoma, and stomach adenocarcinoma, among others (Figure 6). Missense mutations are particularly observed in the peptidase transcript (Figure 7) (72). As proven remarkably by the team of doctor Kevin A. Janes (23), maturation of bioactive GDF11 is defective in TNBC due to insufficient PCSK5 activity but, as shown, the frequency of mutations appear not to be related with the flaw (Figure 8). Once again, transcriptional and post-translational regulation should be considered in future research.

## Effects of GDF11 as Metabolism Regulator

The impact of GDF11 in the development of pancreas implies that the growth factor could exert some metabolic regulation on this organ in the adult, particularly in the endocrine pancreas (67). Dichmann and coauthors found that in the gdf11^−/−^ mouse, the maturation and number of β- and α-cells are normal, however, another group led by Harmon reported that the gdf11^−/−^ mouse exhibited impairment maturation of β-cells and an increment in α-cells, which could produce glucagon in comparison with the wild type mouse (68). This controversy, which is not unusual, must be addressed, but makes it clear that GDF11 could be inducing effects in the metabolism mediated by the pancreas.

Recently, a work by Anon-Hidalgo et al. (73) reported a convincing study associating the circulating levels of GDF11 with thyroid-stimulating hormone (TSH) in humans. The study showed subjects with high or normal levels of TSH present high level contents of GDF11, compared with patients with low levels of TSH. This finding could be due to the fact that other members of the family, such as GDF8 and GDF15, are regulators of the energy homeostasis (74, 75). Anon-Hidalgo team states that it could be related to a regulation of TSH by GDF11, or GDF11 could be positively regulated by TSH or any other thyroid hormones (73).

Luo et al. published that GDF11 decreased lipid content in human mesenchymal stem cells and the mouse 3T3-L1 cell line. This was associated with the repression of adipogenic genes, such as the transcription factors *Pparg, Cebpa*, and the executer proteins *Plp, Cd36, Plin1, Adipoq*, among others, in a mechanism associated to the canonical signal transduction mediated by SMAD2/3 (76). The report provides evidence that GDF11 could exert control over lipid content in unclear fashion. The role of GDF11 in lipid homeostasis could be directed to lipid uptake or efflux, intervening in lipogenic or lipolysis pathways, or lipid removal by autophagy, but data provided by Luo et al. suggest an intervention in lipogenesis. Interestingly, obese mice fed with a high lipid diet present significantly decreased circulating GDF11 levels, compared with mice under low fat diet (77). The mRNA and protein content of GDF11 in skeletal muscle from mice under the high fat diet correlated with the serum content of the growth factor, exhibiting lower expression and protein content, compared with animals under low fat diet. Furthermore, palmitate treatment in the mouse-derived myoblast cell line, C2C12, decreases GDF11 expression. However, the GDF11 did not ameliorate the palmitate-induced insulin resistance and GDF11 treatment did not change expression of Glut4 or Irs-1.

The evidence sustains the metabolic intervention by GDF11, at least in terms of lipid homeostasis, and again in cells with stemness features. This could be relevant in cancer, since lipid overload is one of the main characteristics required for a proper cancer cell proliferation (78, 79). In fact, it is reported that GDF11 impairs mitochondrial function in cancer cell lines, particularly in HCC-derived cells (14). The impact of GDF11 in the central metabolic organelle could explain the tumor suppressive properties exerted by the growth factor. Mitochondria provide essential intermediaries required for cell proliferation: driving redox and calcium homeostasis, coordinating energy supply and mediating cell survival; all of which are fundamental for all cells, and particularly for transformed ones (80). A report by Hernandez-Rizo and collaborators states that GDF11 restricts cell proliferation in hepatic tumor cells through glycolysis and lipid metabolism impairment (81). In agreement with these findings, Garrido-Moreno et al. (82) recently reported that GDF11 prevents cardiomyocyte hypertrophy by preserving the communication between the mitochondria and sarcoplasmic reticulum and calcium mobility, preserving oxidative mitochondria metabolism by a mechanism mediated by the maintenance of mitochondrial cytosolic calcium buffering capacity.

Although the evidence of GDF11 regulation of the energetic and lipid metabolism is limited, it clearly indicates an effect tending to maintain the cellular energetic homeostasis. More research is required to characterize the mechanism underlying metabolic regulation by the growth factor, particularly in cancer cells.

## Concluding Remarks and Future Prospective

GDF11 is an intriguing non-conventional growth factor, perhaps the most fascinating new member of the TGF-β superfamily. It transduces, as practically all members, by the canonical SMAD and non-canonical MAPK pathways, but its functions can be quite variable, even contradictory, depending of the cell lineage, tissue (Table 1), or even age. This raises a complex body of physiological control, which could also differ in health or disease. GDF11 displays a versatile response that must be fully characterized, due to it representing an interesting point of intervention in many diseases or physiological conditions, particularly in cancer. It is remarkable that one of the main characteristics in GDF11 target cells, in normal or pathological conditions, is the stemness capacity. The effects exerted by the growth factor in cancer have begun to be characterized with greater scientific rigor and mechanistic approaches.

**Table 1:** Overview of cancer cell lines or tissue from patients with differential effect of GDF11, as tumor suppressive or tumor promotion protein.

**Cancer**	**Cell/Tissue**	**Tumor suppressive**	**Tumor promotion**	**References**
Liver	Huh7 Hep3B SNU-182 Hepa1-6 HepG2	X		(14)
Liver	HepG2 SMMC-7721 and tissue	X		(60)
Breast	MDA-MB-231 MDA-MB-468 and tissue	X		(23)
Breast	MCF-7	X		(63)
Pancreas	PANC-1 CFPAC-1 Tissue	X		(65)
Colorectal	Tissue		X	(56)
Colorectal	Tissue		X	(70)
Colorectal	CACO-2		X	(69)
Oral squamous cell carcinoma	Tissue		X	(57)
Uveal melanoma	Tissue		X	(71)

Perhaps it is time that GDF11, due to its diverse functionality, constitutes its own subfamily as an atypical and versatile member of the TGF-β family.

We must be cautious to oversimplify its functions. The controversies found clearly indicate that GDF11 displays particular activities depending of cell type, grade of differentiation, and pathological or normal conditions. This remarkable atypical member of the TGF-β family must be carefully studied in clear and well-controlled biological systems. The knowledge, regarding GDF11, will surely be increased in the next few years. The mechanism of action in each particular cancer or cell type must be elucidated to clarify these controversies, and perhaps they will stop being such, thanks to the mechanistic enlightenments obtained in the incoming research in the field.

## Author Contributions

AS-N, MG-R, GP-V, and LC-R conception and preparation of the manuscript. LB, VS, and RM-L reviewed and corrected the manuscript. MCG-R and LG-Q final review of the manuscript and financial support.

### Conflict of Interest

The authors declare that the research was conducted in the absence of any commercial or financial relationships that could be construed as a potential conflict of interest.
